# Survey on the usage and frequency of digital magnification devices in dentistry

**DOI:** 10.1186/s12903-026-07935-1

**Published:** 2026-02-18

**Authors:** Emine Kitin, Oktay Yazicioglu, Abdullah Pekgoz, Sera Gulhan Kuzucan

**Affiliations:** 1 Department of Restorative Dentistry, Faculty of Dentistry, Istanbul Health and Technology University, Istanbul, Turkey; 2 Department of Restorative Dentistry, Faculty of Dentistry, Istanbul University, Istanbul, Turkey; 3 Private Dentmetric Oral and Dental Health Polyclinic, Istanbul, Uskudar Turkey

**Keywords:** Dental magnification devices, Dental education, Dental loupes, Ergonomics, Cross-sectional survey

## Abstract

**Objective:**

This study aimed to evaluate the awareness, usage frequency, and perceptions of dental magnification devices (DMDs) among academic dental practitioners.

**Methods:**

A cross-sectional survey was conducted at the Istanbul University Faculty of Dentistry, which targeted academic staff, doctoral candidates, and specialization students. A total of 159 participants completed an 18-item multiple-choice questionnaire. The data were collected via email and QR codes and statistically analyzed via descriptive and inferential methods.

**Results:**

The results revealed increasing awareness and usage of DMDs, with ergonomic benefits and improved treatment precision being the most commonly cited advantages. Despite these benefits, barriers such as cost and concerns over device dependency have limited broader adoption. The participants indicated that magnification was particularly valuable in endodontics and restorative procedures.

**Conclusion:**

The findings emphasize the growing recognition of DMDs in academic dental practice and highlight the need to incorporate magnification training into dental education curricula to enhance clinical skills and ergonomic awareness from an early stage.

**Supplementary Information:**

The online version contains supplementary material available at 10.1186/s12903-026-07935-1.

## Introduction

Magnification devices found their place in the medical field in the late 19th century and gradually became integral to dental practice. The first microscope specifically designed for dentistry was developed by Apotheker and Jako in 1978, marking the start of magnification use in modern dental care [1].

Today, magnification tools such as dental loupes and microscopes are widely used across clinical dentistry [2–4]. Loupes typically provide magnification in the range of 2.5×–6.5×, whereas microscopes allow higher levels, often up to 40×, particularly in procedures requiring extreme precision [3, 5]. Their main benefits include improved visualization, compensation for near-vision deficiencies, and the promotion of proper posture, which helps prevent musculoskeletal disorders commonly associated with dentistry [6]. Loupe systems are commonly classified as flip-up or through-the-lens (TTL), with further subdivisions such as Galilean or prismatic designs. Each type offers distinct ergonomic and optical properties, making them suitable for different clinical and educational settings [6, 7]. Microscope systems, while less common due to cost and learning curve, provide the highest magnification and depth of field [4].

Previous studies in different countries have examined the prevalence, benefits, and barriers of dental magnification devices. For example, Farook et al. reported cost-related challenges among trainers and trainees in the UK, Eichenberger et al. emphasized the importance of early adoption of optical aids to compensate for visual deficiencies in their Swiss study, while Penmetsa et al. highlighted affordability as a major barrier in India [3, 4, 8]. Similarly, Ferreira et al. demonstrated high usage rates of magnification tools among endodontists in Brazil [9]. Despite this growing body of international research, there remains a significant gap in understanding the adoption and perception of these devices within the Turkish dental academic community, leaving a notable absence of comprehensive data from Turkish dental institutions.

This study therefore aims to address these gaps by investigating the usage patterns, perceived benefits, and barriers of magnification devices among specialists, doctoral students, and faculty members at Istanbul University, one of Turkey’s largest dental faculties. The findings will not only contribute to the international literature on dental ergonomics but also provide valuable insights for curriculum development and institutional policy regarding magnification technology in dental education.

## Materials and methods

This survey study was conducted with the approval of the Clinical Research Ethics Committee of the Faculty of Dentistry at Istanbul University (2023/52 REV-1). This cross-sectional study was conducted and reported in accordance with the STROBE (Strengthening the Reporting of Observational Studies in Epidemiology) guidelines for observational studies. A completed STROBE checklist has been included as a supplementary file to ensure transparent and comprehensive reporting of the study methodology and findings.The study included students pursuing specialization or doctoral degrees and faculty staff at the Faculty of Dentistry, Istanbul University. Undergraduate dental students were excluded from the study.

The survey was distributed to a target population of 204 participants via email through the faculty secretariat. Additionally, a QR code linked to the survey was shared directly with accessible participants to facilitate online completion. Out of 204 potential participants, 159 completed the survey, yielding a response rate of 77.9% (159/204). Individuals who could not be reached, did not respond, or failed to complete the survey were excluded from the analysis. No imputation methods were used for missing data.

The survey consisting of 18 multiple-choice questions was developed through a systematic process. The initial questionnaire items were adapted from previously published studies, particularly those by Farook et al. and Aboalshamat et al., which had established reliability in assessing dental magnification device usage [3, 10]. The questionnaire was then modified with additional items specific to our research objectives. To ensure content validity, the draft questionnaire was reviewed by two experienced faculty members in Restorative Dentistry who assessed the relevance, clarity, and comprehensiveness of the items. Based on their feedback, necessary modifications were made to improve the questionnaire’s face and content validity.

Following expert review, a pilot test was conducted with 15 participants (including 5 doctoral students, 5 specialization students, and 5 faculty members) who were not included in the final study sample. The pilot test aimed to assess the clarity of questions, identify any ambiguities, and evaluate the time required to complete the survey. Feedback from the pilot test indicated that all questions were clearly understood, and no further modifications were necessary. The average completion time was approximately 5–7 min.

The final questionnaire included items on demographic information, previous experience with magnification devices, frequency of use, types of devices used, sources of information about magnification devices, perceived barriers to use, advantages and disadvantages, and application in various dental specialties. Responses were collected via an online platform, recorded, and subsequently analyzed statistically. 

The sample size for this study was calculated via the G*Power 3.1.7 statistical analysis program. The study’s power was expressed as 1-ß (where ß represents the probability of a Type II error). On the basis of the effect size (d) of 0.3976437 derived from the study by Farook et al. [3], a minimum of 145 participants was required to achieve 95% power at an α level of 0.05.

Simple descriptive statistics, including frequency and percentage values, were used to represent significant differences between categorical variables. The chi-square test was applied to analyze relationships among categorical data, with a significance level set at *p* < 0.05.

## Results

In study, the data were collected via a survey method and distributed to a target population of 204 participants. The study employed convenience sampling to recruit participants including students and academic staff from the Faculty of Dentistry at Istanbul University. This non-probability sampling technique was selected due to its practicality in accessing the target population of dental professionals and students within the institutional setting. However, individuals who could not be reached, did not respond, or failed to complete the survey were excluded from the analysis. No imputation methods were used for missing data. With a response rate of 77.9%, a total of 159 participants, including specialization and doctoral students as well as academic staff, were included in the study. This sample size exceeded the minimum required sample of 145 participants as determined by our power analysis, ensuring adequate statistical power for the study.Among the participants, 65 were male (40.9%), and 94 were female (59.1%). The demographic data of the participants are presented in Table 1.

**Table 1:** Demographic data of survey participants

Demographic Data		*n*	%
	Female	94	59.1%
Gender Distribution	Male	65	40.9%
	Total	159	100%
Age Range	18–24	26	16.4%
	25–34	87	54.7%
	35–44	17	10.7%
	45–54	13	8.2%
	55–64	14	8.8 %
	> 65	2	1.3%
Academic Title	Professor	10	6.3%
	Associate Professor	18	11.3%
	Assistant Professor (Dr.)	15	9.4%
	Specialization Student	42	26.4%
	Doctoral (PhD) Student	66	41.5%
	Other	8	5%
Years of Professional Experience	Less than 5 years	83	52.2%
	5–10 years	31	19.5%
	10–15 years	13	8.2%
	More than 15 years	32	20.1%

The majority of participants were between 25 and 34 years of age, and more than half had less than five years of professional experience. Doctoral students and specialization students constituted the largest subgroups, reflecting the academic setting of the study.

Overall, approximately half of the respondents reported having used dental loupes, though only a minority used them consistently. Loupes were more common than microscopes, with Galilean loupes preferred over prismatic types. Most participants learned about magnification devices through colleagues or peers, indicating high general awareness.

Cost emerged as the most significant barrier to ownership, with more than four-fifths of participants citing it as the primary limitation. Other disadvantages included concerns about dependence, though ergonomic benefits and improved treatment quality were widely acknowledged. Endodontics and restorative dentistry were identified as the specialties where magnification was most useful.

Despite strong awareness and positive attitudes toward magnification, device ownership remained relatively low. While many participants believed that magnification could improve treatment quality, opinions were divided on whether it increased or reduced procedure time.

Regarding perceived benefit by specialty, respondents most frequently identified endodontics (%83,6) and restorative dentistry as the primary areas where magnification is most useful, with oral and maxillofacial surgery cited next; prosthodontics and periodontology received moderate endorsement, whereas pediatric dentistry, oral diagnosis and radiology, and orthodontics were less frequently named, and only a very small minority considered magnification unimportant in any specialty.

Detailed distributions of responses for each survey item are presented in Table 2.

**Table 2:** Survey questions and detailed data of answers

Survey Questions		n	%
Have you ever used a magnification device before?	Yes	78	49.1 %
	No	81	50.9 %
(Single Response)			
What is the frequency of your use of digital magnification devices?	Always	5	3.1 %
	Usually	21	13.2 %
	Rarely	25	15.7 %
	Tried it for experimental purposes	48	30.2 %
	Never used	60	37.7 %
(Single Response)			
Which types of magnification devices have you used?	Galilean Loupe (2.5x-3.5x)	56	35.2 %
	Combined Magnifier	14	8.8 %
	Prismatic Loupe (1.5x-6x)	40	25.2 %
	Surgical Operating Microscope (40x)	19	11.9 %
	I have not used any of them	69	42.8 %
(Multiple Response)			
From which sources have you obtained information about magnification devices?	From textbooks	52	32.7 %
	From colleagues or friends	101	63.5 %
	From hands-on training	53	33.3 %
	From social media	60	37.7 %
	From academic articles	48	30.2 %
	I have no knowledge about loupes	22	13.8 %
(Multiple Response)			
What are the barriers to the use of magnification devices?	Cost	133	83.6 %
	Better performance without magnification	9	5.6 %
	Magnification devices make no difference in clinical outcomes	4	2.5 %
	I do not trust digital magnification devices	4	2.5 %
	I do not use them due to health issues	10	6.2 %
	Other	20	12.6 %
(Multiple Response)			
What are the perceived advantages of using digital magnification devices?	Visual comfort	128	80.5 %
	Improved treatment quality	95	59.7 %
	Time efficiency	50	31.4 %
	Reduction in muscle pain	69	43.4 %
	None of the above	6	3.7 %
	Other	10	6.3 %
(Multiple Response)			
What are the perceived disadvantages of using digital magnification devices?	Difficulties in visual measurement	53	33.3 %
	Neck and shoulder pain	28	17.6 %
	No disadvantages of dental magnification devices	16	10 %
	Lower back pain	19	11.9 %
	Becoming dependent on the device	93	58.5 %
	Pain in hands and wrists	19	12 %
	Other	18	11.3 %
(Multiple Response)			
In which dental specialties do you believe digital magnification devices are most beneficial?	Not important in any specialty	2	1.3 %
	Orthodontics	6	3.8 %
	Oral Diagnosis and Radiology	9	5.7 %
	Oral and Maxillofacial Surgery	66	41.5 %
	Restorative Dentistry	102	64.2 %
	Endodontics	133	83.6 %
	Prosthodontics	46	28.9 %
	Periodontology	42	26.4 %
	Pedodontics	20	12.6 %
(Multiple Response)			
Do you follow innovations in digital dentistry?	Yes	124	78 %
	No	8	5 %
	Undecided	27	17 %
(Single Response)			
Do you believe that magnification devices such as dental loupes and microscopes facilitate restorative dental procedures and enhance treatment quality?	Yes	139	87.4 %
	No	5	3.1 %
	Undecided	15	9.4 %
(Single Response)			
Do you think the use of digital magnification devices increases the duration of dental procedures?	Yes	68	42.8 %
	No	53	33.3 %
	Undecided	38	23.9 %
(Single Response)			
What is your reason for purchasing a digital magnification device?	I considered it necessary for treatment	28	17.8 %
	It was recommended to me	18	11.5 %
	I tried it and liked it	27	17.2 %
	Ergonomics	24	15.3 %
	I do not own the device	106	67.5 %
	To improve the quality of my work	1	0.6 %
(Multiple Response)			
What is your reason for not purchasing a digital magnification device?	I do not consider it necessary for treatment	18	11.5 %
	I find the prices too high	92	59 %
	I do not find it aesthetic or practical	4	2.6 %
	I tried it and did not like it	6	3.8 %
	I have purchased and own the device	37	23.7 %
	Other	16	10.3 %
(Multiple Response)			
Do you believe that technological devices facilitate dental procedures?	Yes	148	93.1 %
	No	1	0.6 %
	Undecided	10	6.3 %
(Single Response)			

The inferential statistical analysis revealed significant associations between demographic characteristics and magnification device usage. Usage differed significantly by gender (p = 0.025), with males reporting higher ever-use than females. Professional experience was also significantly associated with adoption (p = 0.023), with mid-to-late career dentists—particularly those with 10–15 years in practice—reporting greater use than early-career colleagues. Adoption varied significantly by age (p = 0.022), with higher uptake among dentists aged 35–44 and 45–54 relative to younger cohorts. In contrast, academic title did not reach statistical significance for either binary use (p = 0.084) or usage frequency (p = 0.077). Perceptions that magnification improves treatment quality differed significantly by experience (p = 0.016), with more experienced dentists more likely to endorse this benefit. Chi-square test statistics examining associations between demographic characteristics and magnification device usage patterns and perceptions are summarized in Table 3.

**Table 3:** Chi-square analyses of associations between categorical variables

Variable	Outcome measure	χ²	df	*p* value
Gender	Ever-use of magnification	5.01	1	0.025
Professional experience (years)	Ever-use of magnification	9.55	3	0.023
Age group	Ever-use of magnification	13.19	5	0.022
Academic title	Ever-use of magnification	9.70	5	0.084
Academic title	Frequency of magnification use	29.59	20	0.077
Professional experience (years)	Perception that magnification improves treatment quality	10.26	3	0.016

A two-sided *p* value < 0.05 was considered statistically significant

## Discussion

In dentistry, manual dexterity and visual acuity are critical for success. Magnification devices are tools utilized in dental practice, which require precise visual capabilities for working on teeth and soft tissues. These devices facilitate a transition from the conventional macro dentistry approach to a more precise micro dentistry methodology [11]. Today, dentists can choose from a wide range of magnification systems, including simple single-lens telescopic magnifiers, compound prismatic telescopic lenses, and various surgical microscopes [12]. This study achieved a 77.9% response rate among 159 participants (65 male, 94 female), with gender distribution closely matching Istanbul University Faculty of Dentistry’s 2023 admission ratios (59.8% female, 40.2% male).

The majority of participants in this study were doctoral students, who demonstrated a positive level of awareness regarding magnification devices. This reflects the tendency of doctoral students to engage more actively in academic research and scientific studies, showing a greater willingness to participate in such endeavors. Similarly, Karl et al. highlighted the increasing use of digital magnification devices in dentistry. Their study emphasized that digital magnification tools could play a pivotal role in the education and practical training of young clinical dentists [13].

Nearly half of the participants reported prior use of dental loupes, indicating their widespread preference as tools in dental practice. However, the proportion of nonusers is also noteworthy, suggesting that some dentists have yet to adopt this technology or may prefer alternative tools. Braga et al. emphasized the significant role of dental loupes in dental practice, highlighting that their use during preparation exercises on models led to improved outcomes among dental students [14]. Similarly, Ferreira et al., in their study involving 279 endodontists in Brazil, reported a 67.3% usage rate of dental microscopes and loupes in dental practice [9]. Conversely, Topkara et al. reported that only 14.2% of practitioners used magnification devices during endodontic treatment, which is a notably lower rate. This disparity may be attributed to regional and economic factors [15].

The widespread use of dental loupes can be attributed to several factors, such as ergonomic benefits and enhanced visual accuracy during surgical procedures. Brown et al., in their study on the advantages of dental loupes, specifically emphasized their ergonomic benefits and the ease they provide in surgical applications [16]. However, the high proportion of nonusers suggests that dental loupes may not suit certain clinical or personal preferences or that specific barriers may hinder their use. Low et al. identified challenges such as difficulty adapting to a new working style, the high cost of magnification devices and their accessories, additional infection control requirements, and potential risks of injury from sharp instruments during procedures as reasons why dentists might avoid using magnification systems [17]. A recent study also emphasizes that high cost and lack of training are the major factors limit the usage of dental magnifiying devices [18]. These findings underscore the need to promote the adoption of dental loupes and encourage nonusers to benefit from this technology.

Colleagues and peers were identified as the primary source of information on magnification devices, which reflects the strong role of professional networks in shaping clinical decisions. This reliance on interpersonal exchange is consistent with previous studies, such as Eichenberger et al., who noted that recommendations from colleagues were more influential than other factors in purchase decisions [4]. Social media and other channels played a secondary but still notable role, suggesting that multiple sources contribute to awareness. The high overall level of knowledge about loupes in our sample likely reflects both these diverse information pathways and the increasing visibility of magnification in dental training and practice.

A small but notable proportion of respondents indicated no knowledge of digital magnification devices. This highlights a gap in exposure that is particularly concerning within an academic setting, where future practitioners are being trained. The absence of awareness underscores the importance of integrating magnification topics into undergraduate curricula, ensuring that all students—regardless of their specialization or level of training—receive early and consistent education on the clinical and ergonomic benefits of these tools. The incorporation of such topics into dental education could help future practitioners understand and utilize these technologies effectively.

In the survey, a considerable number of participants indicated that they had never used digital magnification devices, reflecting limited exposure despite their recognized benefits. Cost was reported as the most significant barrier to the adoption of magnification devices, with over four-fifths of participants citing financial constraints. This finding is in line with previous research highlighting the economic burden of purchasing loupes. For example, Penmetsa et al. reported that high expense was a key limiting factor for practitioners in Andhra Pradesh, India, while Farook et al. found that cost remained a considerable challenge for dental trainers and trainees in the UK [3, 8]. As in these contexts, our results emphasize that affordability is a critical determinant of loupe adoption. However, the financial constraints observed in our study are particularly specific to Istanbul University, where both students and faculty face additional limitations related to local income levels and the relatively high cost of imported devices. Addressing this challenge may require institutional interventions, such as bulk procurement, subsidies, or providing shared access to magnification devices during training, thereby reducing the individual financial burden and ensuring early exposure to their ergonomic and clinical benefits.

In line with the findings of Alhazzazi et al., the majority of participants in our study reported that the use of magnification devices improved treatment quality, providing an additional advantage in clinical practice [19]. The ease of using magnification devices depends on the chosen system. Among the participants, Galilean loupes were preferred by 35.2%, surpassing the use of surgical microscopes. This preference may stem from their lightweight design and ease of use, particularly at the introductory stage. Jain et al. similarly recommended lightweight and user-friendly magnification devices for initial use [20].

Most participants used 2.5x–3.5x magnifying Galilean loupes and 1.5x–6x magnifying prismatic loupes for routine dental procedures, whereas surgical microscopes with up to 40x magnification were employed for surgical procedures. Consistent with the findings of Christensen, magnification devices play a significant role in dental practices, providing valuable guidance in determining appropriate levels of magnification [7].

The participants generally reported that magnification devices are beneficial because of improvements in treatment quality and the resulting time savings during procedures. Similarly, research has demonstrated that enhanced treatment quality, combined with the use of microsurgical instruments to minimize tissue trauma, significantly accelerates healing processes [21].

While few participants believed that digital magnification devices have no disadvantages, the most frequently cited concern among those who noted drawbacks was the risk of dependency (58.5%). This finding requires nuanced interpretation, as it may reflect either negative perception or positive adaptation to higher clinical standards. From one perspective, this concern could indicate apprehension about losing fundamental clinical skills or becoming overly reliant on technology [17].

However, from an ergonomic standpoint, this ‘dependency’ may actually represent beneficial adaptation to improved working conditions and enhanced visual acuity standards. Ergonomics is one of the most critical principles in dental practice, and research in dental ergonomics consistently demonstrates that practitioners who use magnification devices develop superior postural habits and experience reduced musculoskeletal strain [22, 23]. Hong et al. (2024) demonstrated that microscope use significantly reduced muscle workload in the neck and shoulder muscles during crown preparation procedures while Costa et al. confirmed their role in lowering the risk of musculoskeletal disorders [24, 25]. Hayes et al. (2016) conducted a comprehensive study on the effect of loupes on neck pain and disability among dental hygienists, finding that while no statistically significant differences were detected, wearing loupes appeared to have both positive and negative outcomes with regards to physical well-being, suggesting the need for further long-term studies [26]. Valachi and Valachi (2003) demonstrated that magnification use promotes better neck positioning and reduces the risk of cervical spine disorders, suggesting that practitioners who feel uncomfortable working without these devices may have developed healthier working patterns [27]. The ergonomics literature supports the view that consistent magnification use leads to improved clinical outcomes and reduced operator fatigue [28]. These findings support our results, in which participants widely acknowledged ergonomic benefits as one of the key advantages of magnification devices. In this context, reluctance to work without magnification may indicate positive ergonomic conditioning rather than problematic dependency.

Furthermore, the concept of ‘dependency’ in magnification use parallels the adoption of other essential clinical tools. Just as practitioners become ‘dependent’ on proper lighting or high-quality instruments, reliance on magnification may represent professional evolution toward higher precision standards rather than a limitation [6]. This interpretation is particularly relevant in our sample, where financial constraints often restrict device access - the fear of dependency may be heightened by concerns about inconsistent availability rather than the technology itself. Therefore, what participants perceive as ‘dependency’ may actually reflect the natural progression toward evidence-based ergonomic practices. This finding suggests that educational interventions should reframe magnification use as professional advancement rather than technological dependence, emphasizing the long-term benefits for both practitioner health and patient care quality.

The majority of participants agreed, in line with previous studies, that dental magnifiers are most critical in endodontic and restorative treatments. These findings suggest that participants considered magnification particularly important in fields where fine detail, precision, and enhanced visibility are critical to clinical success. By contrast, specialties such as pedodontics and orthodontics were less frequently associated with magnification, reflecting the differing visual and technical demands of these disciplines. Penmetsa and Mani similarly reported that the use of dental loupes in restorative and endodontic treatments enhances treatment success [8]. A large proportion of participants (80%) shared the view that dental loupes improve visual comfort, enhance precision, and increase treatment quality, which aligns with findings from other studies. De Oliveira et al. (2024) found that improved restorative preparation performance in 66.6% of cases compared to unaided vision, though higher magnifications showed no additional benefits over lower ones [29]. Similarly, Chourasia et al. (2024) compared unaided vision, 3x loupes, and 7.5x microscope for direct composite restorations, finding microscope use significantly reduced adjacent sound enamel scratching (*p* = 0.022) [30]. A multi-centered study conducted by Alzar et al. demonstrated that a significant proportion of participants acknowledged the positive impact of dental magnification on treatment prognosis and procedural quality, particularly in operative and endodontic treatments [31]. These findings support the routine use of magnification devices in restorative dentistry, particularly for tissue preservation during precise procedures. However, in other studies some certain limitations were also noted, such as challenges during the adaptation period, the additional weight of the magnification system, difficulties in patient positioning, and potential nausea or vertigo triggered by eye movements between magnified and unmagnified visual fields [32–34].

The role of technological devices in simplifying dental procedures is undeniable. A significant majority of participants agreed that technological advancements facilitate clinical workflows. In many countries today, postgraduate programs are designed around new tools and techniques. As reflected in the findings of this study, magnification devices such as dental loupes and microscopes not only provide convenience to practitioners but also enhance the quality of dental treatments.

The frequency of digital magnification device usage in dentistry has been widely debated in the literature, with numerous studies addressing this topic. A review of these articles further deepens the discussion on the adoption of digital magnification devices in dental practice. The advantages and disadvantages of these devices should be assessed in alignment with the individual needs and practice styles of each practitioner.

Given these factors, it is reasonable to predict a growing trend in the use of digital magnification devices in dental practice, with their adoption becoming even more widespread in the future.

A notable strength of this study is its relatively high response rate (77.9%), which reduces the risk of non-response bias and increases the reliability of the findings. In addition, the sample included a wide range of academic positions, from doctoral students and specialization students to professors and associate professors. This diversity provides valuable insight into how magnification devices are perceived and used across different levels of professional experience and academic seniority. The study also addresses an underexplored population in Turkey, thereby contributing original data to the international literature on dental ergonomics and magnification use.

Unlike previous surveys that primarily documented usage patterns, our findings reveal a nuanced paradox specific to academic dental settings in developing economies. The high awareness (> 85%) combined with low consistent usage (< 20%) suggests that barriers are not rooted in knowledge deficits but rather in institutional and economic constraints. This awareness-ownership paradox represents a novel finding that challenges the assumption that education alone drives technology adoption in dental practice. These findings have important implications for dental education and policy. Early integration of magnification devices into undergraduate and postgraduate curricula could help normalize their use and foster ergonomic habits at the beginning of clinical training. Furthermore, institutional strategies—such as providing shared access in teaching clinics, offering subsidies, or negotiating bulk procurement—may reduce the financial burden on individuals and promote wider adoption. Despite high awareness, device ownership and regular use remained low, indicating an “awareness–ownership paradox” driven by structural barriers (cost and access) and underscoring the need for institutional procurement and training policies to resolve this paradox.

A limitation of this study is that the sample was restricted to a single institution, Istanbul University, and included a relatively modest number of participants. While the response rate was high, these factors limit the external validity of the findings. Therefore, the results should be interpreted with caution and may not be generalizable to all dental schools or practitioners across Turkey or internationally.

## Conclusion

This survey identified a clear discrepancy between awareness and ownership of digital magnification devices among dental practitioners at Istanbul University. Although the majority of participants demonstrated strong knowledge of loupes and microscopes, only a minority owned or consistently used them. The principal barrier to adoption was cost, reflecting both the local economic context and limited institutional support.

Unlike previous surveys that primarily reported on prevalence or user attitudes, this study highlights a context-specific paradox: high awareness combined with low ownership. This nuance emphasizes that barriers are not rooted in lack of knowledge but rather in economic and institutional constraints, suggesting that solutions must extend beyond individual decision-making.

These findings have important implications for dental education and policy. Early integration of magnification devices into undergraduate and postgraduate curricula could help normalize their use and foster ergonomic habits at the beginning of clinical training. Furthermore, institutional strategies—such as providing shared access in teaching clinics, offering subsidies, or negotiating bulk procurement—may reduce the financial burden on individuals and promote wider adoption. Addressing these gaps will not only increase accessibility but also ensure that future dentists benefit from the visual and ergonomic advantages of magnification in daily practice.

Given the single-institution design and limited sample size, these findings should be interpreted with caution and validated through larger, multi-center studies that could further explore this awareness-ownership paradox in different economic and institutional contexts in the future.

## Supplementary Information

Supplementary Material 1.

## Data Availability

The data that support the findings of this study are available upon request from the corresponding author. The data are not publicly available due to privacy or ethical restrictions.

## References

[1] Apotheker H, Jako GJ. A microscope for use in dentistry. J Microsurg. 1981;3(1):7–10. 7341737 10.1002/micr.1920030104

[2] Meraner M, Nase JB. Magnification in dental practice and education: experience and attitudes of a dental school faculty. J Dent Educ. 2008;72(6):698–706. 18519600

[3] Farook SA, Stokes RJ, Davis AK, Sneddon K, Collyer J. Use of dental loupes among dental trainers and trainees in the UK. J Investig Clin Dent. 2013;4(2):120–3. 23097188 10.1111/jicd.12002

[4] Eichenberger M, Perrin P, Ramseyer ST, Lussi A. Visual acuity and experience with magnification devices in Swiss dental practices. Oper Dent. 2015;40(4):E142–149. 25748209 10.2341/14-103-C

[5] Karaca İR, Gündoğdu M. Diş hekimiğinde kullanılan büyütme sistemleri: Derleme. Ortadoğu Tıp Dergisi. 2018;10(3):374–80.

[6] James T, Gilmour AS. Magnifying loupes in modern dental practice: an update. Dent Update. 2010;37(9):633–6. 21179934 10.12968/denu.2010.37.9.633

[7] Christensen GJ. Magnification in dentistry: useful tool or another gimmick? J Am Dent Assoc. 2003;134(12):1647–50. 14719762 10.14219/jada.archive.2003.0111

[8] Penmetsa GS, Mani LP, Praveen G, Dwarakanath CD, Suresh S. Awareness, Attitude, and prevalence of usage of magnification devices among the dental practitioners in the state of Andhra Pradesh–A questionnaire-based study. J Indian Soc Periodontology. 2017;21(5):398–402. 10.4103/jisp.jisp_268_17 PMC5827508 29491587

[9] Ferreira ACG, Frozoni M, Prado M, Gomes B, Signoretti F, De-Jesus-Soares A. Current trends in technological armamentarium and treatment among Brazilian endodontists. Brazilian J Oral Sci. 2017;16:1–10.

[10] Aboalshamat K, Daoud O, Mahmoud LA, Attal S, Alshehri R, Bin Othman D, Alzahrani R. Practices and attitudes of dental loupes and their relationship to musculoskeletal disorders among dental practitioners. Int J Dent. 2020;2020(1):8828709. 32802059 10.1155/2020/8828709 PMC7414349

[11] Mallikarjun SA, Devi PR, Naik AR, Tiwari S. Magnification in dental practice: how useful is it? J Health Res Reviews (In Developing Countries). 2015;2(2):39–44.

[12] Gupta P, Jan SM, Behal R, Mir RA, Shafi M, Teli ZA. Periodontal microsurgery: a review. IOSR J Dent Med Sci. 2014;13(4):12–7.

[13] Karl E. Dental students’ performance and perceived experience with magnifying dental loupe. Open Access J Dent Oral Surg (OAJDOS). 2023;4:1–4.

[14] Braga T, Robb N, Love RM, Amaral RR, Rodrigues VP, de Camargo JMP, Duarte MAH. The impact of the use of magnifying dental loupes on the performance of undergraduate dental students undertaking simulated dental procedures. J Dent Educ. 2021;85(3):418–26. 32984960 10.1002/jdd.12437

[15] Topkara C, Özyürek T, Demiryürek EÖ, Bursalı T, Özler M. Attitudes, materials, and methods preferred in root Canal treatment in turkey: a survey. Priv Pact. 2017;142(2):51–6.

[16] Brown M, Qualtrough A, Mclean W. Magnification in undergraduate endodontic teaching in the UK and ireland: a survey of teaching leads in endodontology. Int Endod J. 2020;53(4):553–61. 31644836 10.1111/iej.13240

[17] Low JF, Dom TNM, Baharin SA. Magnification in endodontics: A review of its application and acceptance among dental practitioners. Eur J Dent. 2018;12(4):610–6. 30369811 10.4103/ejd.ejd_248_18 PMC6178675

[18] Alghilan MA, AlShehri A, Almeshrafi A, Alrumi A, Aldibasi O. Decision-Making factors among dentists for using dental magnifying loupes: A Cross-Sectional study. Clin Cosmet Investig Dent. 2025;17:99–110. 39927158 10.2147/CCIDE.S501104 PMC11807348

[19] Alhazzazi TY, Alzebiani NA, Alotaibi SK, Bogari DF, Bakalka GT, Hazzazi LW, Jan AM, McDonald NJ. Awareness and attitude toward using dental magnification among dental students and residents at King Abdulaziz University, faculty of dentistry. BMC Oral Health. 2016;17(1):21. 27430209 10.1186/s12903-016-0254-4 PMC4950692

[20] Jain R, Kudva P, Kumar R, Nagar S. Periodontal microsurgery-magnifying facts, maximizing results. J Adv Med Dent Sci Res. 2014;2(3):24–34.

[21] Burkhardt R, Hürzeler MB. Utilization of the surgical microscope for advanced plastic periodontal surgery. Pract Periodontics Aesthet Dent. 2000;12(2):171–80. quiz 182. 11404959

[22] Lindegård A, Nordander C, Jacobsson H, Arvidsson I. Opting to wear prismatic spectacles was associated with reduced neck pain in dental personnel: a longitudinal cohort study. BMC Musculoskelet Disord. 2016;17:347. 27535742 10.1186/s12891-016-1145-1 PMC4989289

[23] Aboalshamat K, Daoud O, Mahmoud LA, Attal S, Alshehri R, Bin Othman D, Alzahrani R. Practices and attitudes of dental loupes and their relationship to musculoskeletal disorders among dental practitioners. Int J Dent. 2020;2020:8828709. 32802059 10.1155/2020/8828709 PMC7414349

[24] Hong S, Park J, Jeon M-J, Shin S-J, Park JH, Park J-W. Effect of loupe and microscope on dentists’ neck and shoulder muscle workload during crown Preparation. Sci Rep. 2024;14(1):17489. 39080435 10.1038/s41598-024-68538-w PMC11289447

[25] Costa RT, Miranda SB, Montes MA, Ribeiro AK, Carreiro AF, Moraes SL. Impact of using magnifying dental loupes on clinical performance during tooth preparation: A systematic review. J Clin Exp Dent. 2024;16(2):e186–97. 38496818 10.4317/jced.61098 PMC10943671

[26] Hayes MJ, Osmotherly PG, Taylor JA, Smith DR, Ho A. The effect of loupes on neck pain and disability among dental hygienists. Work. 2016;53(4):755–62. 26890593 10.3233/WOR-162253

[27] Valachi B, Valachi K. Mechanisms leading to musculoskeletal disorders in dentistry. J Am Dent Assoc. 2003;134(10):1344–50. 14620013 10.14219/jada.archive.2003.0048

[28] Bowers DJ, Glickman GN, Solomon ES, He J. Magnification’s effect on endodontic fine motor skills. J Endod. 2010;36(7):1135–8. 20630285 10.1016/j.joen.2010.03.003

[29] de Oliveira FAS, Moraschini V, de Almeida DCF, Dos Santos GO. Effects of magnification on restorative dental Preparation performance: a scoping review and level of evidence mapping. Clin Oral Investig. 2024;28(8):447. 39052037 10.1007/s00784-024-05852-7

[30] Chourasia HR, Nandalur KR, Daghrery A, Vinothkumar TS, Khormi Y, Tawhari AI, Kariri WH. The effect of magnification on the quality of direct posterior composite restorations and the adjacent sound enamel: an in vitro study. Eur J Gen Dentistry. 2024;14(02):154–60.

[31] Iqbal A, Karobari MI, Shrivastava D, Srivastava KC, Arjumand B, Algarni HA, Alonazi MA, Alnasser M, Khattak O, Syed J, et al. Practices and preferences in the use of magnification among endodontists and restorative dentists: A multicentre study. PLoS ONE. 2025;20(1):e0311391. 39775371 10.1371/journal.pone.0311391 PMC11706448

[32] Sunell S, Rucker L. Surgical magnification in dental hygiene practice. Int J Dental Hygiene. 2004;2(1):26–35. 10.1111/j.1601-5037.2004.00061.x 16451449

[33] Sunell S, Maschak L. Positioning for clinical dental hygiene care. Preventing back, neck and shoulder pain. Probe (Ottawa Ont). 1996;30(6):216–9. 9611451

[34] Thomas J, Thomas FD. Dental hygienists’ opinions about loupes in education. Am Dent Hygienists’ Association. 2007;81(4):82–82. 18173896

