# Supplementary material for: Survey on the usage and frequency of digital magnification devices in dentistry
Source: BMC Oral Health. 2026 Feb 18;26:528. doi: 10.1186/s12903-026-07935-1 (PMC13020365; doi:10.1186/s12903-026-07935-1)
Supplement: Supplementary file 1 — Supplementary Material 1. [file 12903_2026_7935_MOESM1_ESM.docx]

**STROBE checklist for Survey on the Usage and Frequency of Digital Magnification Devices in Dentistry**

| **Item No** | **Recommendation** | **Page No** | **Check** |
| --- | --- | --- | --- |
| **Title and abstract** | |  |  |
| 1 | (a) Indicate the study's design with a commonly used term in the title or the abstract (b) Provide in the abstract an informative and balanced summary of what was done and what was found | 1 | ✓ |
| **Introduction** | |  |  |
| **Background/rationale** | |  |  |
| 2 | Explain the scientific background and rationale for the investigation being reported | 1-2 | ✓ |
| **Objectives** | |  |  |
| 3 | State specific objectives, including any prespecified hypotheses | 2 | ✓ |
| **Methods** | |  |  |
| **Study design** | |  |  |
| 4 | Present key elements of study design early in the paper | 2 | ✓ |
| **Setting** | |  |  |
| 5 | Describe the setting, locations, and relevant dates, including periods of recruitment, exposure, follow-up, and data collection | 2-3 | ✓ |
| **Participants** | |  |  |
| 6 | (a) Give the eligibility criteria, and the sources and methods of selection of participants | 2-3 | ✓ |
| **Variables** | |  |  |
| 7 | Clearly define all outcomes, exposures, predictors, potential confounders, and effect modifiers. Give diagnostic criteria, if applicable | 3 | ✓ |
| **Data sources/measurement** | |  |  |
| 8 | For each variable of interest, give sources of data and details of methods of assessment (measurement). Describe comparability of assessment methods if there is more than one group | 3 | ✓ |
| **Bias** | |  |  |
| 9 | Describe any efforts to address potential sources of bias | 3 | ✓ |
| **Study size** | |  |  |
| 10 | Explain how the study size was arrived at | 3 | ✓ |
| **Quantitative variables** | |  |  |
| 11 | Explain how quantitative variables were handled in the analyses. If applicable, describe which groupings were chosen and why | 3 | ✓ |
| **Statistical methods** | |  |  |
| 12 | (a) Describe all statistical methods, including those used to control for confounding (b) Describe any methods used to examine subgroups and interactions (c) Explain how missing data were addressed (d) If applicable, describe analytical methods taking account of sampling strategy (e) Describe any sensitivity analyses | 3 | ✓ |
| **Results** | |  |  |
| **Participants** | |  |  |
| 13 | (a) Report numbers of individuals at each stage of study—eg numbers potentially eligible, examined for eligibility, confirmed eligible, included in the study, completing follow-up, and analysed (b) Give reasons for non-participation at each stage (c) Consider use of a flow diagram | 3-6 | ✓ |
| **Descriptive data** | |  |  |
| 14 | (a) Give characteristics of study participants (eg demographic, clinical, social) and information on exposures and potential confounders (b) Indicate number of participants with missing data for each variable of interest | 3-6 | ✓ |
| **Outcome data** | |  |  |
| 15 | Report numbers of outcome events or summary measures | 3-7 | ✓ |

*Note: This checklist was adapted from the STROBE Statement guidelines (von Elm E, Altman DG, Egger M, Pocock SJ, Gøtzsche PC, Vandenbroucke JP; STROBE Initiative. The Strengthening the Reporting of Observational Studies in Epidemiology (STROBE) statement: guidelines for reporting observational studies. J Clin Epidemiol. 2008;61(4):344-9.)*
